# Comprehensive Evaluation of Gene Expression in Negative and Positive Trigger-based Targeting Niosomes in HEK-293 Cell Line

**DOI:** 10.22037/ijpr.2019.112058.13507

**Published:** 2020

**Authors:** Mahmood Barani, Masoud Torkzadeh-Mahani, Mohammad Mirzaei, Mohammad Hadi Nematollahi

**Affiliations:** a *Department of Chemistry, Institute of Nanochemistry, Shahid Bahonar University of Kerman, Kerman, Iran. *; b *Department of Biotechnology, Institute of Science, High Technology and Environmental Sciences, Graduate University of Advanced Technology, Kerman, Iran. *; c *Physiology Research Center, Institute of Basic and Clinical Physiology Sciences, Kerman University of Medical Sciences, Kerman, Iran. *; d *Department of Biochemistry, School of Medicine, Kerman University of Medical Sciences, Kerman, Iran.*

**Keywords:** Gene expression, Targeting, Niosome, Magnetic nano particle, Plasmid

## Abstract

An efficient gene delivery system has some critical factors that enhance the efficiency of nanocarrier. These factors are low production cost, high bioavailability, high encapsulation efficiency, controllable release, and targeting ability. Niosome (the nonionic surfactant vesicles) was considered as a promising gene delivery system. Niosome can increase stability and uptake of active agents. We used all mentioned factors in one optimized formulation entitled plasmid- loaded magnetic niosomes (PMN). To increase the bioavailability of niosomes, we used ergosterol (a natural lipid) instead of cholesterol in structure of niosome. Also, cetyl trimethyl ammonium bromide (CTAB) in different concentrations was used to improve encapsulation of plasmid and compared to niosomes that did not have CTAB (negative niosome). Afterward, magnetic nanoparticle (Fe_3_O_4_@SiO_2_) was synthesized and loaded into niosome to obtain targeting ability. Prepared formulations were evaluated regarding size, zeta potential, morphology, encapsulation of magnetic nanoparticles and plasmid (Pm-cherry-N1), release rate, and transfection efficiency. Results demonstrated that optimum formulation (Nio/CTAB3%/Fe/P) has a nanometric size (118 ± 2.31 nm, positive zeta potential (+25 ± 0.67 mV), high loading of plasmid (72%), and good gene expression (35%). Interestingly, after applying a magnetic field below the cell plate, we obtained ac increased gene expression from 35% to 42%. These results showed that this new formulation would have a promising future and also can be used for delivering the other drugs and active agents.

## Introduction

Gene delivery is based on introducing a specific DNA to the target cell to cure some genetic disorders (1). The clinical application of gene-based therapy for treating some diseases has been investigated (2). However, *in-vivo* delivery of genes to the target cell is a significant challenge (3-5). Moreover, entry of DNA into cells and enzymatic digestion are challenging problems in gene delivery (6). Generally, viral and non-viral carriers are used in gene delivery. Non-viral vectors have found many applications in gene therapy because of their low immunogenicity, high accumulations and also controlled the release of the entrapped gene (7). Non-viral vectors can be polymer (8), liposome (9-12), niosome (13, 14), peptide, dendrimer (15), and MOF (Metal Organic Framework) (16). Among these carriers, niosomes (spherical and bioavailable non-viral vectors that made of non-ionic surfactants) have promising future for gene delivery purposes (17-19), owing to their unique structural characteristics such as higher storage time, low cost synthesize, more stability, simpler surface modification, lower toxicity, and thus higher bioavailability than others.

Most niosome formulations in gene delivery applications have some critical parts: a) non-ionic surfactants (20), b) cholesterol or its derivatives as helper lipid (21-26), and c) an additive that serves as positive charge inducer and increases interaction between niosome and plasmid and influences the transfection efficiency and toxicity (27). Cetyl trimethyl ammonium bromide (CTAB) is a positively charged molecules, commonly used as a cationic additive for preventing aggregation of niosomes and in this case (gene delivery), enhances interaction between niosome and plasmid (28). Ergosterol, a major fungal membrane sterol, has been utilized as helper lipid instead of cholesterol. The chemical structure of this sterol is similar to cholesterol, but their tail sections are different from each other. Ergosterol can interact more effectively with non-ionic surfactants because its tail has a double bond (25, 29). There is no report about niosome stabilized by ergosterol as a gene delivery vector.

Magnetic nanoparticles have some benefits for targeting, such as increasing contrast in magnetic resonance imaging technique, producing non-toxic ions when they degrade in *in-vivo* conditions, and the most important, directing them to the specific site by a magnetic field (30-35). For example, magnetic liposomes (liposomes with entrapped magnetic particles) are so attractive because they encapsulate drug into a protecting shield and at the same time can deliver the drug to the target cell (36-38). The first magnetic liposome was reported in the 1990s accumulated at the target tissue by an external magnetic field (39-42). Anyway, a few studies have reported the application of magnetic niosomes in gene targeting (43, 44).

In this study, we prepared magnetic niosomes based on Span 60, Tween 60, and ergosterol. We hypothesized maybe because of a double band in the tail of ergosterol, it can interact more effectively with the surfactants of niosome and make a more stable formulation. This stability means there is no much free surfactant for cytotoxicity of the cells. Also, CTAB as a cationic agent was used to give positively charged niosomes and was compared with niosomes without CTAB, that had a negative zeta potential. To achieve a targeted gene delivery vector, we synthesized silica-coated magnetic nanoparticles (Fe_3_O_4_@SiO_2_) and incorporated them along with Pm-cherry-N1 plasmid model into niosomes. Many authors mentioned that coating of Fe_3_O_4_ with silica could improve the bioavailability of the magnetic nanoparticles (45).

This novel carrier system has been characterized in terms of size, zeta potential, magnetic particle content, gene entrapment efficiency, and gene release rate. Also, we used MTT assay for cytotoxicity evaluation of formulations. To evaluate the magnetic vesicles as a gene targeting carrier, *in-vitro* assay was done by an external magnetic field that was placed below the culture plate. Till now this is the first report that uses ergosterol as helper lipid for preparation of cationic and anionic magneto-niosomes for gene delivery.

## Experimental

*Materials*

Polysorbate-60 (Tween 60), Span 60, Ergosterol (Ergo), and 3-(4, 5-dimethylthiazol-2yl)-2, 5-diphenyltetrazolium bromide (MTT reagent) were purchased from Sigma (St. Louis, MO, USA). Ferrous chloride tetrahydrate (FeCl_2_.4H_2_O), ferric chloride hexahydrate (FeCl_3_.6H_2_O), sodium hydroxide, tetraethylorthosilicate (TEOS), and Cetyl trimethyl ammonium bromide (CTAB) were obtained from Merck (Germany). PicoGreen® dsDNA Quantitation Reagent and Kits were purchased from Invitrogen (Carlsbad, California, US). pDNA was amplified in the Escherichia coli strain DH5α and purified using a QIAGEN Plasmid Giga Kit (QIAGEN, Hilden, Germany). For *in-vitro* experiment, 0.4 T (Tesla) neodymium magnet was used.

*Magnetic Fe nanoparticles with silica shell (Fe*_3_ *O*_4_ *@SiO*_2_ *)*

At the first stage, the co-precipitation method was used for Fe_3_O_4_ magnetic nanoparticles (46). Prepared nanoparticles washed several times with alcohol and water. Then, Fe_3_O_4_@SiO_2_ was prepared by the coating of SiO_2_ shell on the Fe_3_O_4_ core. Briefly, 25 mL of 10% TEOS was added to 50 mL of the Fe suspension and mixed by a heater stirrer. pH was set at 9.0 with NaOH solution and the obtained solution heated to 90 °C and stirred for 8 h. Washing procedure with water and ethanol was done five times. In the end, the final suspension was held at a cool place until next use (47).

*Preparation of niosome containing Fe*_3_ *O*_4_ *@SiO*_2_

For the preparation of niosome, we used thin film hydration method (48, 49). At the first, stock solutions of each niosome component at a concentration of 50 mg/mL in chloroform was prepared. Then, Span 60, Tween 60, and ergosterol at molar ratios of 35:35:30 were added to an RB flask (50 mL). The solvent was removed by vacuum rotary (Laboroa 4003, Heidolph, Germany) at 60 °C, 120 rpm, and 120 min. After evaporation of chloroform, Hydration of the obtained thin film was performed with 5 mL of PBS solution (pH 7.4) of plasmid (1 mg/mL) along with Ferrofluid solution (6.5 × 10^−7^M) and 3 and 5 W/V% of CTAB at 60 °C for 30 min and 180 rpm. After this procedure, the prepared formulations incubated overnight for plasmid inclusion. Then, the formulations were sonicated in an ultrasonic bath for 25 min to small uni-lamellar vesicles be achieved (50). Untrapped plasmids and magnetic nanoparticles were separated from entrapped ones by centrifuging at 15000 rpm for 15 min and 23 °C (5415D, Eppendorf, Germany). For niosome filtration, the formulations passed through the 400 nm and then 200 nm membrane filter pore sizes (BIOFIL Syringe Filter, China).

*Characterization of silica-coated magnetic nanoparticles*

The crystal structure of as-prepared Fe_3_O_4_@SiO_2_ was analyzed by X-ray diffraction (XRD) (Panalytical, Almelo, Netherlands). The molecular structure of Fe_3_O_4_@SiO_2_ was investigated by a Fourier transform spectrometer (FTIR, Bruker, Saarbrucken, Germany) at room temperature (25 °C). The magnetic property was measured by a vibrating sample magnetometer (VSM, Danesh Pajohan Kavir Co., Kashan, Iran).

*Dynamic light scattering measurements*

The size, polydispersity index (PDI), and ƺ-potential of the formulations were characterized by dynamic light scattering (DLS) (Malvern, Helix, UK) at 25 °C by measurement of the autocorrelation function at 90°. The average size and standard error (± SD) were measured by the instrument fitting data. Each experiment was carried out in triplicate.

*Morphology*

The morphology of the resulted Fe_3_O_4_@SiO_2_ NPs and niosomes were assessed using a scanning electron microscopy (SEM) (SBC-12, KYKY, China).

*Entrapment efficiency of silica-coated magnetic nanoparticles*

The loading content of magnetic nanoparticles was assessed by the method described in ref (39). Briefly, purified niosomal samples (0.1 mL) were mixed with 0.1 mL of a methanol solution (7%, v/v) and then magnetic materials were ionized by adding 1.5 mL of 2M HCl. Ionized particles were reduced by adding 1.5M of hydroxylamine hydrochloride. For complexation, 11 mM of o-phenanthroline was added, neutralized by 5M of NaOH and pH was kept at 4.5 by citrate buffer. The absorption peak of the prepared complex was read at 510 nm, and the loading efficiency of magnetic nanoparticles in niosomes was calculated.

*Gene entrapment efficiency*

The pmCherry-C1 plasmid encoding Cherry fluorescent protein was used. pDNA encapsulation efficiency was expressed as the percentage of the gene entrapped into the filtered niosomes referred to the total amount of gene present in a non-filtered sample (49). It was quantified by use of a PicoGreen kit by diluting 1 mL of each formulations in 25 mL of methanol, followed by the calculation of absorbance of these solutions at the wavelength of 520 nm by Fluorimeter plate reader (FLx800, BioTek, US), where PicoGreen dye shows a maximum emission peak at this wavelength (51, 52). Methanol breaks the membrane of niosomes and allows the release of the encapsulated gene. Each experiment was carried out in triplicate.

*Electrophoresis assay of DNA in niosome formulations*

Retention of the naked DNA and niosomal samples (containing plasmid) was assessed by 1% gel electrophoresis containing ethidium bromide. The gel was immersed in a buffer containing EDTA, Tris, and Acetate to exposure to a 120 V for 25 min. The bands were observed by a digital imaging system Alliance 4.7 (UVITEC, Cambridge, UK). 

*In-vitro release of plasmid*

Magneto-niosomes containing plasmid were add in a dialysis bag (Spectra/Por®, cut off 12–14 kDa) (53). A solution of 50 mL PBS buffer with pH 7.4 was used to mimic conditions of physiological fluids in the body. At the specific time intervals, the sample quantities were withdrawn and characterized by Fluorimeter plate reader using PicoGreen assay. To guarantee sink conditions, medium amounts equivalent to the removed volumes were added. The results were taken as mean values of three runs.

*In-vitro cytotoxicity assay*

Niosome cytotoxic effect on HEK-293 cells (Invitrogen, Milan, Italy) was evaluated with MTT assay. The MTT assay was carried out according to the protocol described for the first time by Mosmann (54). The assay was optimized for the cell line used in these experiments. Briefly, HEK-293 cells (1 × 10^4^) in 100 µL of either medium alone or medium containing formulations at concentrations of 5 to 30 µM were added to each well of a 96-well plate (Costar, Charlotte, NC). The plate was maintained at 37 °C in a 5% CO2 atmosphere for 24 h. MTT (15 µL, 4 mg/mL) was then added to each well. After incubation for further 4 h, DMSO (100 µL, 0.520 mM) was added to each well for solubilizing formazan dye. Then the absorbance of the control and niosome-treated wells was measured by using plate reader (FACSCalibur, Beckton Dickinson, US) at a wavelength of 490 nm. The cytotoxicity, C (%), was calculated as follows:

C% = (1 - (A (nt))/A (C)) × 100 

Where A (nt) and A (C) are, respectively, the absorbance of niosome-treated and control well. Values were expressed as the mean of three different experiments ± SD.

*Gene expression*

HEK-293 cell was seeded in 24-well plates at an initial density of 6 × 10^4^ cells/well, with high glucose DMEM containing 10% fetal bovine serum (FBS). Then a defined volume of media was removed, and formulations were added to the cells. After 24 h, the reporter gene (pmCherry-C1) expression was monitored and quantified by Becton and Dickinson flow cytometer (BD Company, Franklin Lakes, NJ). The fluorescent protein was excited at 587 nm and emission was detected using a 610/20 filter. Flow cytometry analysis was performed to the quantitative determination of transfected genes with and without applying an external magnetic field that was placed below the cell plate (0.4 T (Tesla) neodymium magnet and 10 min incubation time) (55).

*Statistical analysis*

One-way ANOVA test was used for statistical analysis of the various experiments. A posterior Bonferroni *t*-test was performed to examine the ANOVA test. A *p*-value < 0.05 was considered statistically significant. 

**Figure 1.**
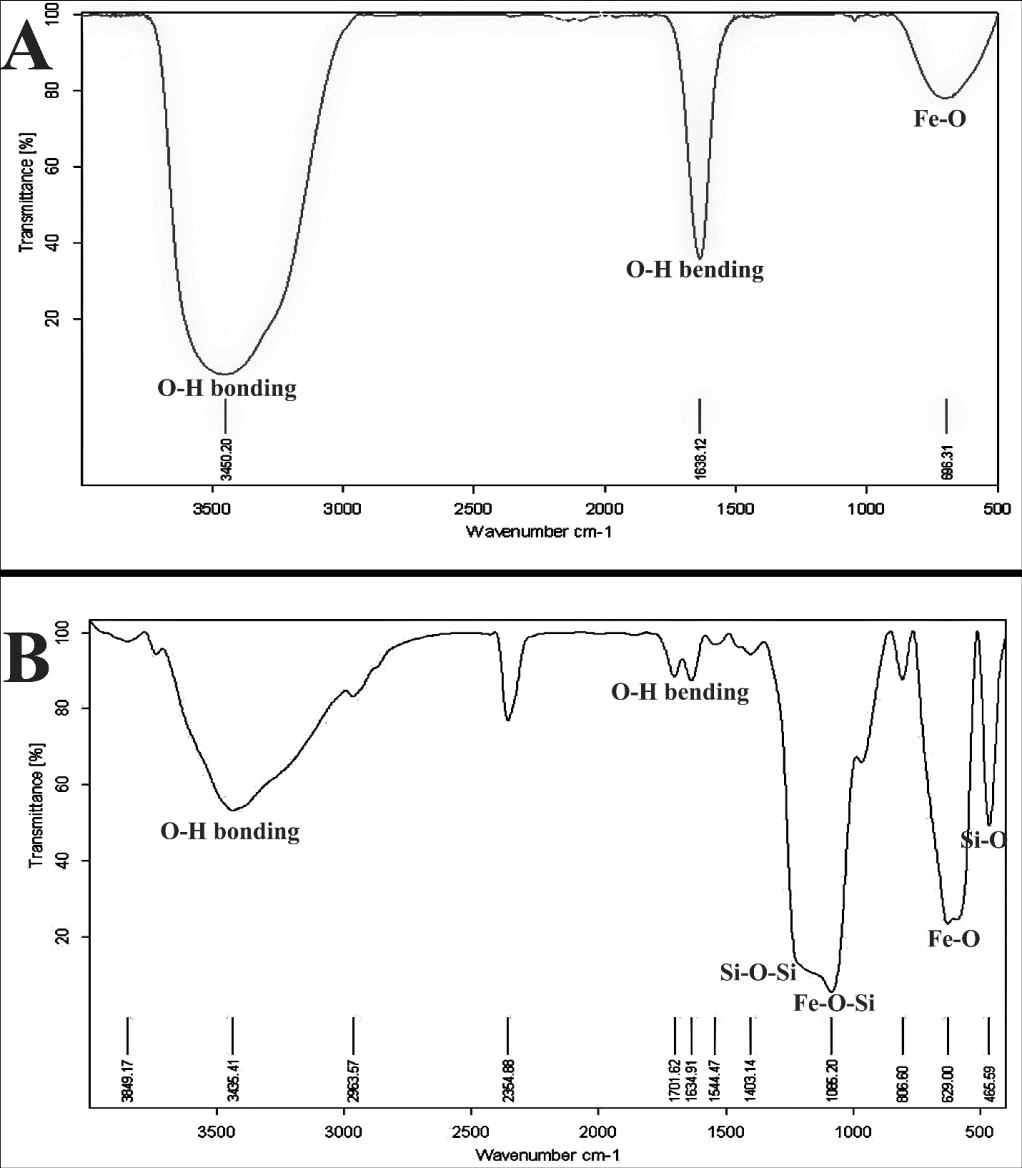
FTIR spectra of (A) Fe_3_O_4_ and (B) Fe_3_O_4_@SiO2 NPs

**Figure 2:**
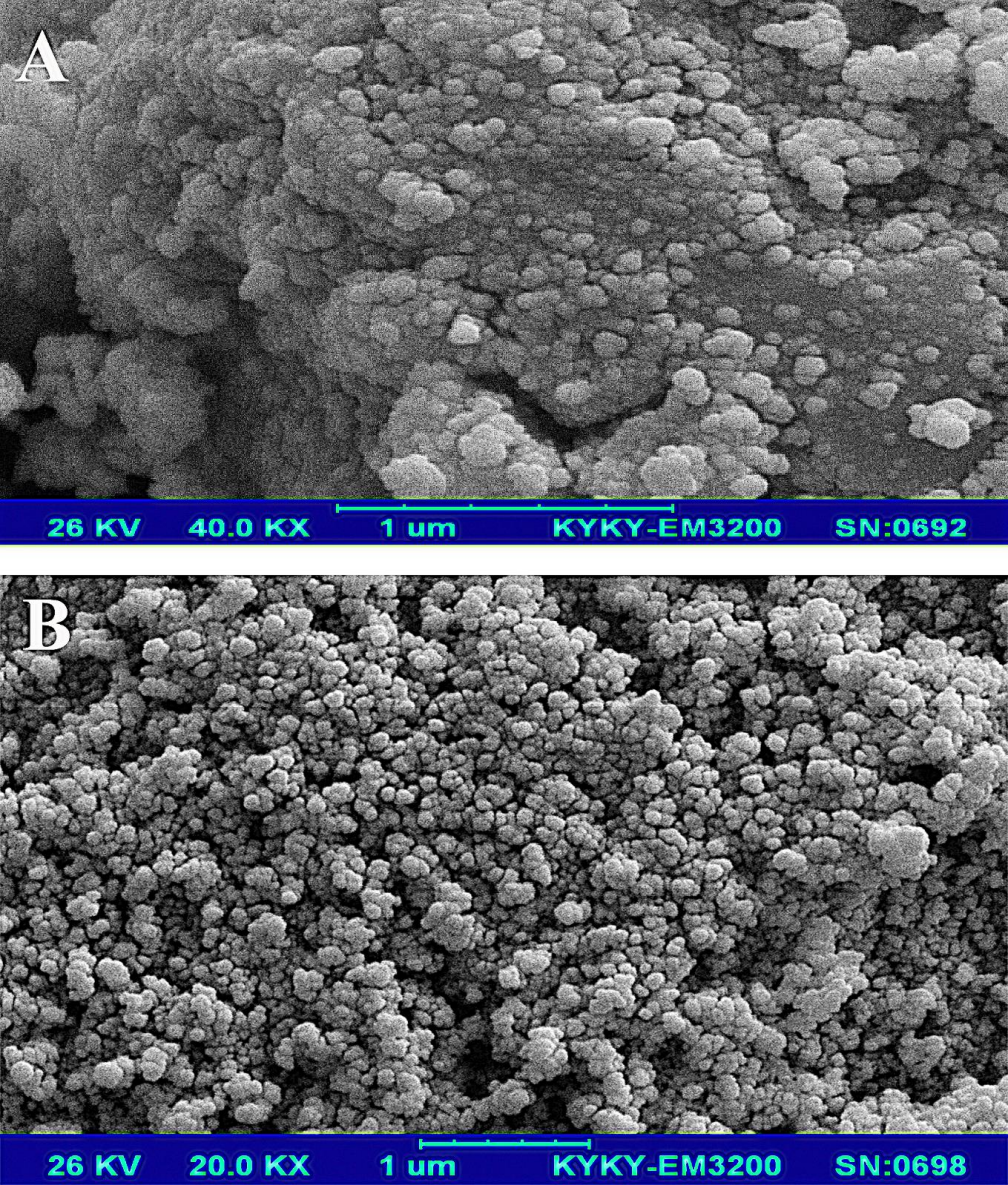
SEM image of (A) Fe3O4 and (B) Fe3O4@SiO2 NPs at different magnifications

**Figure 3:**
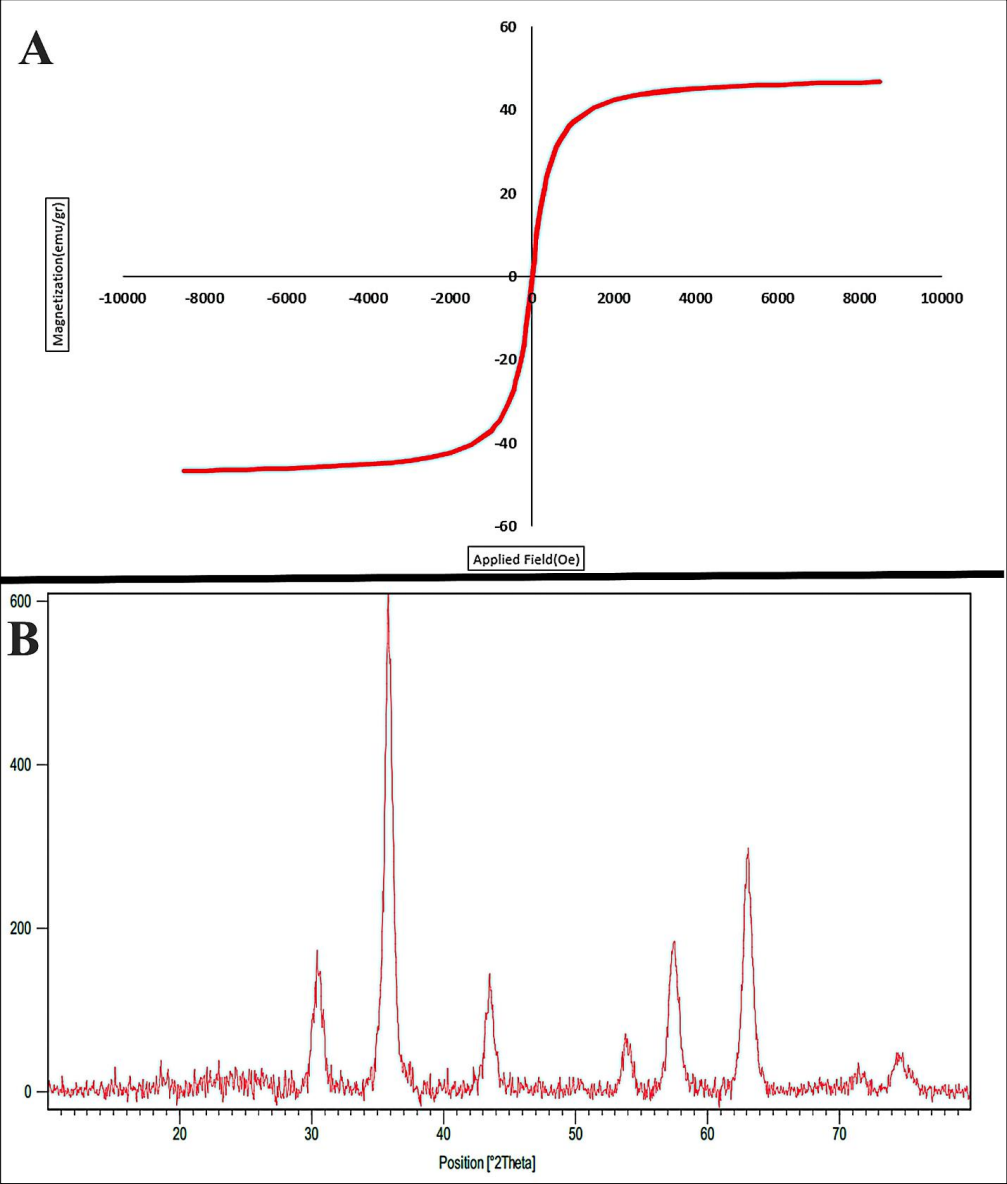
(A) The magnetic behavior (VSM analysis) and (B) XRD pattern of Fe3O4@SiO2 nanoparticles

**Figure 4.**
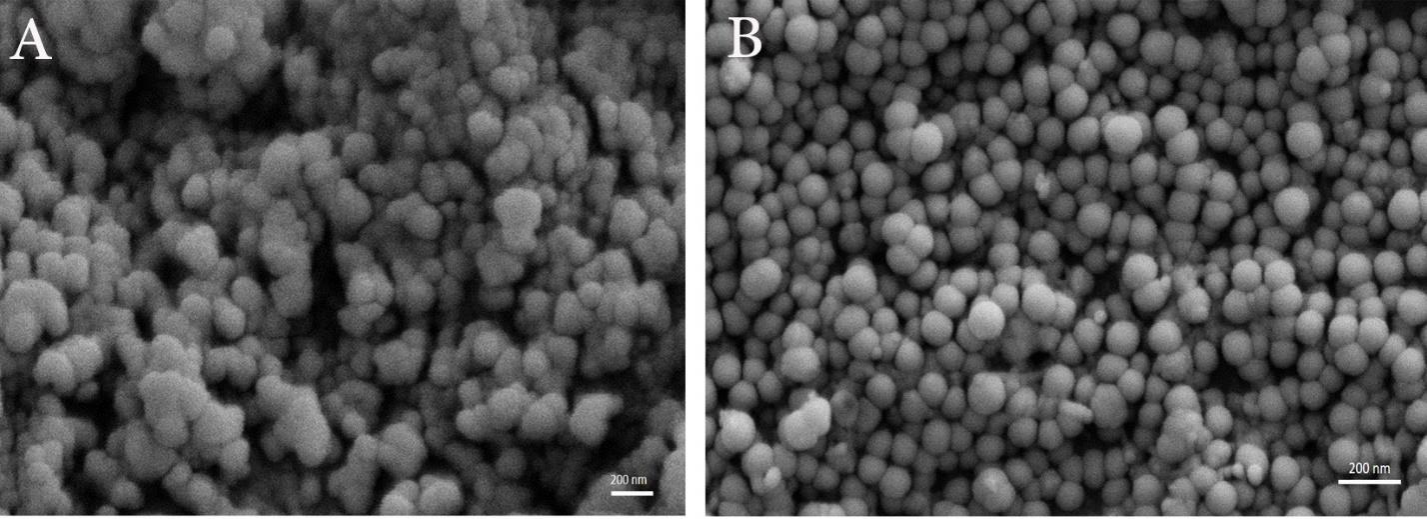
SEM images of formulations. (A) Negative niosomes, (B) Positive niosomes (3%), original magnification 40.000×.

**Figure 5:**
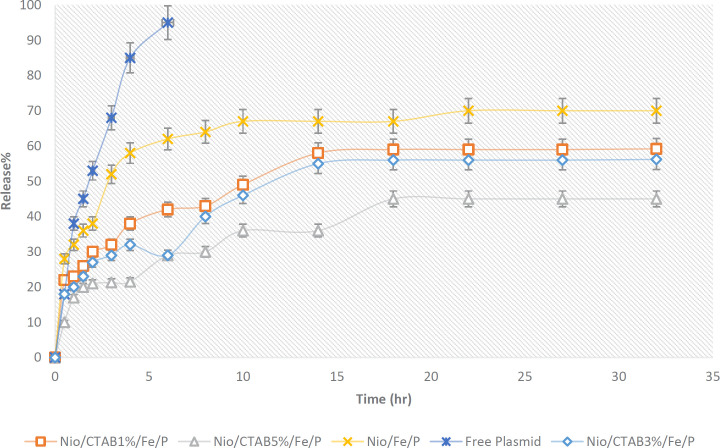
Sustained release (%) of free plasmid and plasmid entrapped in niosomes in phosphate buffer saline (PBS, pH 7.4) at 37 °C. Points, mean (n = 3); bars, SD

**Figure 6:**
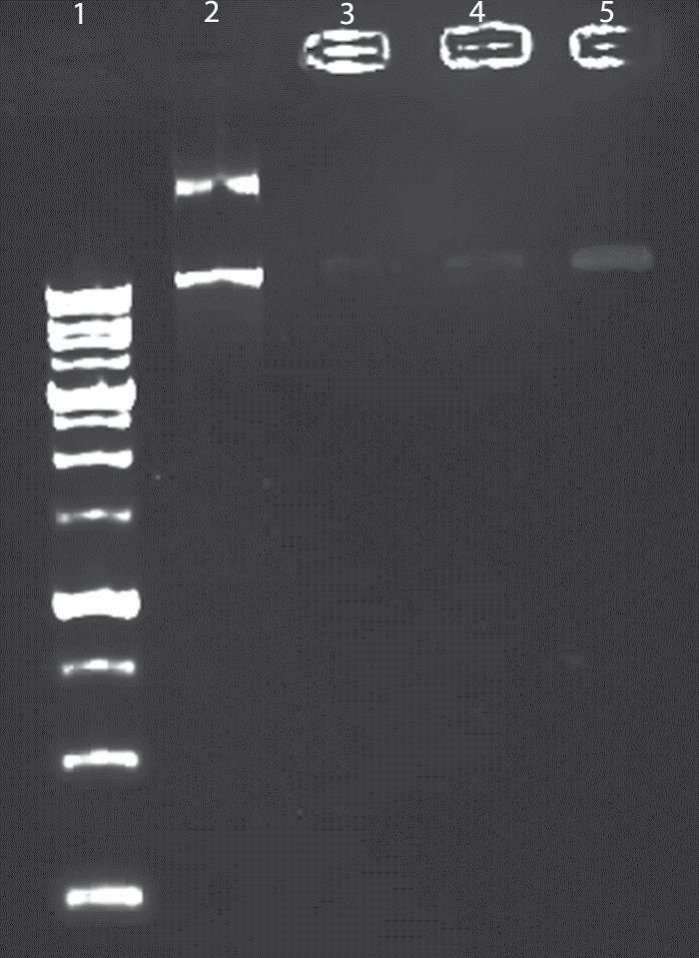
Binding, protection, and DNase-induced release of DNA from niosomes visualized by agarose electrophoresis.; lane 1, ladder; Lane 2 correspond to free DNA; lane 3 Nio/CTAB5%/Fe/P; lane 4; Nio/CTAB3%/Fe/P and lane 5; Nio/Fe/P

**Figure 7:**
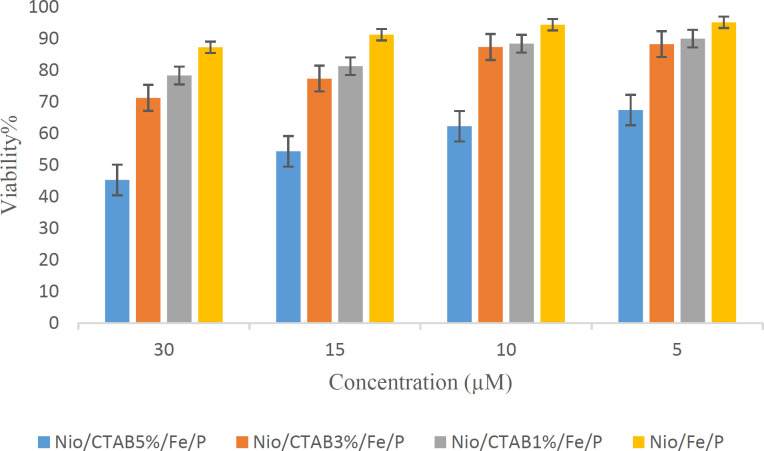
Cell viability of HEK-293 cell line (MTT test) after treatment to different concentrations (5, 10, 15, and 30 µM) of positive and negative-based niosomes loaded with Plasmid (P) and MNPs (Fe)

**Figure 8:**
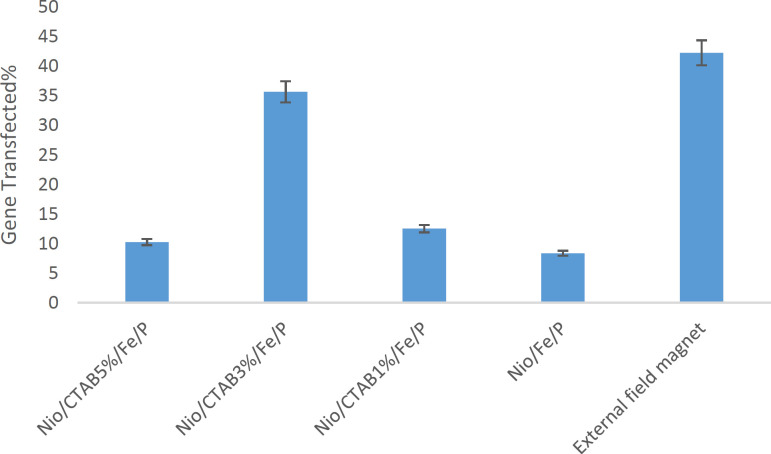
Flow cytometry results of HEK-293 after an 24 h incubation period at 37 °C. Values represent mean ± SD, n = 3

**Table 1:** Composition and physicochemical characterization of niosome formulations

**Name**	**Size (nm)**	**Polydispersity** **index**	**Zeta potential** **(mV)**	**EE% (magnetite** **entrapment)**	**EE%** **(plasmid entrapment)**
Nio/CTAB5%/Fe/P	102 ± 3.32	0.14 ± 0.01	+32 ± 0.25	92	83
Nio/CTAB3%/Fe/P	118 ± 2.31	0.17 ± 0.03	+25 ± 0.67	84	72
Nio/CTAB1%/Fe/P	123 ± 2.57	0.19 ± 0.02	+21 ± 0.67	86	61
Nio/Fe/P	132 ± 1.16	0.21 ± 0.04	-23 ± 0.82	88	39
Nio/Fe	120 ±1.98	0.24 ± 0.05	-21 ± 0.16	91	-
Nio/P	135 ± 2.32	0.23 ± 0.08	-21 ± 0.14	-	57
Nio	125 ± 1.38	0.026 ± 0.05	-18 ± 0.28	-	-

Nio: Niosome; Fe: Fe_3_O_4_@SiO_2_ NPs; P: Plasmid.

## Results and Discussion

*Characterization of magnetic nanoparticles*

*FTIR spectrum*

The characterization of the prepared Fe_3_O_4_ and Fe_3_O_4_@SiO_2_ NPs was surveyed by FTIR spectra. Figures 1A and 1B show the FTIR spectrum of Fe_3_O_4_ and Fe_3_O_4_@SiO_2_ NPs which have some peaks around 696 cm^-1^ assigned to bonding vibrations of Fe-O. The existence of SiO_2_ shell in Figure 1B can be approved by the Si–O–Si stretching vibration and the Fe–O–Si stretching vibration frequencies at 1097 and 1080 cm^−1^, respectively. The obtained data showed that the SiO_2_ layer was formed on the surface of Fe_3_O_4_ magnetic nanoparticles as recently discussed by Luong *et al.* on SiO_2_-coated FePt nanoparticles. Also, the peaks appeared at 1638 cm^-1^, and 3450 cm^-1^ corresponding to H–O–H bending and O–H bonding (hydroxyl groups) vibrations of the nanoparticles, respectively. 

*Scanning electron microscopy*

Morphology and the average size of magnetic nanoparticles were characterized by scanning electron microscopy (SEM). In Figures 2A and 2B, the SEM images of the Fe_3_O_4_ and Fe_3_O_4_@SiO_2_ particles show that these particles have an approximately spherical shape, and the average size for the Fe_3_O_4_ and Fe_3_O_4_@SiO_2_ is about 31 and 42 nm, respectively. Fe_3_O_4_@SiO_2_ NPs have a bigger size and more homogenous morphology because of the SiO_2_ layer. The presence of some bigger particles in the images of Fe_3_O_4_ is related to the agglomeration or overlapping of some smaller particles during the preparation step. Davarpanah *et al.* (2019) also used Fe_3_O_4_@SiO_2_ NPs for targeted delivery of Carboplatin to the cancer cells. They reported that Fe_3_O_4_@SiO_2_ NPs have more homogeneity and also more suspension stability than Fe_3_O_4 _(34).

*VSM*

Figure 3A shows the magnetic curve of Fe_3_O_4_@SiO_2_ nanoparticles measured at room temperature. These nanoparticles show a superparamagnetic property (*i.e.*, no remanence effect) with a high saturation magnetization of 66.1 emu/g. The superparamagnetic property of the synthesized nanoparticles is sufficient for gene targeting purposes.

*XRD pattern*

The X-ray diffraction (XRD) patterns Fe_3_O_4_@SiO_2_ is shown in Figure 3B. In this figure, scattering angles 2ϴ have been crystallized in the cubic system with spinel structure (Fd3mwith lattice size of 8.4000 Å, ICSD card # 01-072-2303). The size of the prepared Fe_3_O_4_@SiO_2_ NPs was investigated via XRD measurement and line broadening of the peak at 2ϴ = 5°-80° using Debye-Scherer Equation (56):

D = 0.94λ/βcosϴ

Where d is the crystallite size, λ is the wavelength of the X-ray source, β is the full width at half maximum (FWHM), and ϴ is Bragg diffraction angle. From Debye-Scherer calculations, the crystalline size of Fe_3_O_4_@SiO_2_ NPs was about 19 nm. From Figure 3B, we can observe that Fe_3_O_4_@SiO_2_ nanoparticles have a high crystalline percent. Gao *et al.* (2011) synthesized Fe_3_O_4_@SiO_2_ by StÖber method. The XRD pattern of their NPs are well indexed to the cubic spinel phase of magnetite (57). Cheng *et al.* (2010) reported that coating of SiO_2_ on Fe_3_O_4_ could increase the size of resulted core-shell nanoparticles while the crystalline structure and magnetic properties did not change significantly (58). Above mentioned studies are in agreement with our results.

*Physicochemical characterization of niosomes*

Table 1 compares particle size, PDI, zeta potential, magnetite, and plasmid entrapment efficiency (EE%) of niosomes composing of ergosterol and different CTAB content that were combined with magnetic nanoparticles (MNPs) and plasmid. Formulations varied in size, PDI and zeta potential depending on the encapsulated materials and concentration of additive used in the bilayer.

Generally, positive niosomes have a smaller size, positive zeta potential and better polydispersity index (PDI) than negative niosomes (Table 1). For example, the size and zeta potential of Nio/Fe/P changed from 132 nm and -23 mV to 102 nm and +32 mV for Nio/CTAB5%/Fe/P. When the concentration of CTAB increased, the zeta potential of the formulations moved to positive zeta potentials that were because of CTAB positive nature. PDI ≤ 0.3 corresponds to an intense and small width peak in size distribution profile of the particles (59). PDI of Nio/CTAB3%/Fe/P was 0.14 that moved to 0.21 for Nio/Fe/P. Addition of MNPs and plasmid has an opposite effect on the size of positive and negative niosomes. MNPs decrease the size of niosome probably because some MNPs placed in the bilayer, and there are favorable interactions between the niosomal matrix and MNPs. These interactions could increase the rigidity of bilayer and hence decrease the size (60, 61). Also, stability study of niosomes after six months via size measurement shows that the size of positive niosomes has changed from 102 nm to 119 nm (*p *> 0.05) whereas negative-niosomes has changed from 132 nm to 176 nm (*p *< 0.05) (results not shown). This achievement is in agreement with the PDI index and proved the higher stability of positive niosomes.

As shown in Figure 4, both formulations have a spherical morphology with the excellent size distribution, but interestingly positive niosomes have a smaller size, more spherical shape, and better dispersity than the negative ones.

The entrapment efficiency of magnetic nanoparticle and plasmid was evaluated by colorimetric analysis and PicoGreen kit, respectively. As shown in Table 1, the entrapment of MNPs for positive and negative formulations is almost similar, but entrapment of the plasmid is different. More entrapment of plasmid for positive niosomes was obtained as a result of functional interaction between plasmid with its negative surface charge and niosomes with positive zeta potential.

As shown in Figure 5, the release behavior of plasmid from niosome formulations had a biphasic pattern so that a first rapid release and then a slow and steady release by the time appeared. Also, the release of free plasmid was very fast as about 95% of it was released within 6 h (*p *< 0.05). The release of the plasmid from positive niosomes was slower than negative niosomes because of more electrostatic interactions between positive niosomes and negatively charged plasmid (*p *> 0.05). Many authors mentioned that controlled release of nanocarrier is a critical parameter for gene delivery systems (62).

*Encapsulation plasmid in formulations*

Figure 6 shows the results obtained in the agarose gel electrophoresis assay (Paya Pajoohesh, EPS 7401, Iran). Lanes 3 and 4 show an opaque band due to a functional interaction between plasmid and positive niosomes. Niosome formulation with 5% CTAB hardly eliminated plasmid. Lane 5 shows negative niosomes that demonstrate a visible band because of the weak interaction between negative niosome and negative plasmid. This data showed that niosome effectively could protect from plasmid against harsh conditions and also the interaction between plasmid and carrier was considerable.

*Viability studies*

The *in-vitro* cytotoxic effect of the niosome formulations were tested using human embryonic kidney cell line (HEK-293). Figure 7 shows the treatment of HEK-293 cells with positive and negative based niosomes at concentrations ranging from 5 to 30 µM for 24 h. No toxic effects were observed on cell growth against HEK-293 cells for negative niosomes (5 and 10 µM), but the cell viabilities were decreased as the concentration of formulation increased to 30 µM (*p *< 0.05). This result indicated that a high concentration of niosomes (≥10 µM) could result in higher cell toxicity to HEK-293 cells. Positive niosomes have more toxicity than the negative ones. When the concentration of CTAB in niosome increased, the toxicity of formulation on cell line enhanced. MNPs did not have any toxicity in all formulations and concentrations. Taken together the present data indicate that positive niosomes have higher cytotoxicity due to the CTAB that has a toxic nature. Chaikul *et al.* (2019) reported a similar behavior for CTAB/Niosomes that CTAB can increase the cytotoxicity of the final formulation (63).

*Gene expression study*

Transfection efficiency of niosome formulations with and without applying an external magnetic field on the HEK-293 cell line was evaluated. Under microscopic examination, HEK-293 cells had a normal morphology at all concentrations and all formulations were tested (Data not shown). Percentage of transfected cells was evaluated by flow cytometry. Free DNA did not any transfection in HEK-293 cell line. As observed in Figure 8, the percentage of the transfected cells changed with the change of formulation. The percentage of the transactions ranged from 8% in Nio/Fe/P to a maximum of 42% in the Nio/CTAB3%/Fe/P with applying an external magnetic field. Nio/Fe/P has the lowest transfection because it has negative zeta potential and there is a low interaction between negative plasmid and negative niosomes (*p *< 0.05). The addition of CTAB to formulation increase transfection so that Nio/CTAB3%/Fe/P formulation has highest transfection about 35% while Nio/CTAB5%/Fe/P has 10% transfection (*p *< 0.05). Decrease in the transfection, in this case, is because of the toxicity of CTAB in high concentrations. The effect of magnetic field on the transfection of Nio/CTAB3%/Fe/P formulation was examined by measuring the transfected cells under magnetic induction. The time of magnetic induction was 10 min, and the transfection efficiency without magnetic induction was measured at the same time. The transfect efficiency with the external magnetic field was 42% (*p *< 0.05). We assumed that because of the external magnetic field, the uptake of magnetic niosome into the cell improved. Zheng *et al.* (2009) prepared a magnetic cationic liposome for gene delivery (MCLs/pDNA) (64). They reported that the transfection efficiency of MCLs/pDNA complexes with a relatively lower concentration of MAG-T (0.75 mg/mL) was the same as that of CLs/pDNA complexes without a magnetic field, but by applying an external magnetic field, transfection efficiency increased about 2.6-fold. This result is in good agreement with our data about our magnetic niosomes.

## Conclusion

In this study, we report a new nanocarrier for targeting gene delivery. Negative and positive magnetic niosomes were physicochemically characterized regarding particle size, morph-ology, surface charge, and release of the plasmid. Results show that as the concentration of CTAB incorporated into the niosomes increased, the zeta potential goes to positive values. *In-vitro* transfection experiments were performed on HEK-293 cells. The low transfection efficiency in negative niosomes is probably due to the negative charge surface of these formulations that decrease interaction between niosome and cell membrane. Nio/CTAB3%/Fe/P formulation with an external magnetic field had the highest transfection (about 42%). Taken together, these magnetic-niosomes are potential delivery formulations for the delivery and treatment of genetic disorders.
